# An Integrative Review of Engineering Ethics Education at Delft University of Technology: Contexts, Assumptions, and Practices

**DOI:** 10.1007/s11948-026-00594-z

**Published:** 2026-04-28

**Authors:** Daniel M. Phoenix, Qin Zhu, Andrea Gammon, Rockwell Clancy

**Affiliations:** 1 Department of Engineering Education, Virginia Tech, Blacksburg, VA USA; 2 Faculty of Technology, Policy, & Management, Delft University of Technology, Delft, Netherlands

**Keywords:** Engineering ethics, Ethics education, Case studies, Historical context, Theoretical assumptions, Instructional practices

## Abstract

Engineering ethics education has undergone significant development across institutions worldwide over the last three decades. While earlier analyses have examined aspects of this development, few integrated accounts have traced the evolution of a leading institution’s approach to engineering ethics education across this period. This paper addresses this gap. It presents an integrative review of engineering ethics education at Delft University of Technology (TU Delft), one of Europe’s most influential institutions in this field. Drawing on peer-reviewed articles, books, book chapters, conference papers, and institutional documents published by TU Delft scholars over three decades, we trace the historical development of the institution’s approach, analyze its core theoretical assumptions and examine its learning goals and evolving pedagogical practices. The review identifies two distinct stages in the institution’s development. It reflects a deepening of learning goals and, most notably, a shift toward experiential and non-cognitive pedagogical approaches. These findings offer a reference point for discussing how ethics should be taught to engineers, how to integrate it into technical curricula, and which kinds of normative stances institutions should adopt in their ethics programs.

## Introduction

Systematic analyses of engineering ethics education programs at four-year higher education institutions with a strong emphasis on STEM (science, technology, engineering and mathematics) have recently flourished in the literature (see Homma et al., 2025; Martin et al., 2021). Notably, unlike in previous periods, these analyses have increasingly included countries beyond the United States, Western Europe, and Australia (see Akudugu & Abagale, 2024; Cheruvalath, 2024; Lategan, 2024; Wei & Yuan, 2024). This trend aligns with recent calls in the literature to adopt an institutional perspective on professional ethics education reforms and to embrace a more holistic approach to understanding engineering students’ moral learning experiences and professional formation (see Polmear, 2025, pp. 353–354). This paper is part of a larger project that investigates how engineering students from three cultural contexts, the United States, Netherlands, and China, develop ethical reasoning and moral intuitions over the course of their four-year programs, and how institutional cultures shape moral development experiences. One of the outcomes of this project is to develop a collection of case studies that highlight engineering ethics education programs, with particular attention to how instructional practices and institutional cultures at these programs influence students’ learning in engineering ethics. This paper focuses on engineering ethics education at one of the project’s major participating institutions: Delft University of Technology in the Netherlands.

TU Delft is widely recognized as a pioneering institution in engineering ethics education in Europe. Not in vain, it has been among the first European technical universities to make such ethics courses compulsory at scale (van de Poel et al., 2001). The university has since sustained a dedicated Ethics and Philosophy of Technology Section that teaches ethics across all engineering programs (Taebi & Kastenberg, 2019). During the last decade, it is now experimenting with innovative teaching approaches. The purpose of this paper is to provide an account on how ethical training for engineers is envisioned and enacted at this institution. Such account provides contextual insights to inform the design of further empirical studies. These include case studies of other institutions for comparison purposes and the analysis of the influence of institutional cultures and curriculum design in students’ ethical reasoning.

Engineering ethics education currently faces two interrelated challenges from within the discipline. The first is developing more responsive teaching methods that move beyond traditional lecture-based formats. The second is accounting for the non-cognitive dimensions of ethical learning—that is, the affective, experiential, and embodied aspects of moral formation that purely cognitive approaches tend to overlook. How institutions respond to these challenges has consequences that extend well beyond the discipline itself. Engineers are increasingly central actors in addressing some of the most pressing societal problems of our time. These include making environmental sustainability and human development compatible, managing the social risks of artificial intelligence, and navigating the ethical complexities of biotechnology. To the extent that engineering must play its part in addressing these challenges, rigorous reviews, analyses, and comparative studies of how institutions envision and enact engineering ethics education are consequential in theory and practice.

This paper proceeds as follows. First, it provides a brief overview of the most currently debated topics in engineering ethics education across the European continent, to which this paper seeks to contribute. Second, it outlines our methodology, emphasizing qualitative literature review methods in engineering ethics. Third, it presents the results of the integrative review across three dimensions: a concise historical account of how engineering ethics education has been conceptualized and institutionalized at TU Delft; the three core theoretical assumptions about the moral status of contemporary technologies underpinning its programs; and the instructional practices of TU Delft’s engineering ethics program, including its learning objectives, teaching methods, and innovative pedagogical approaches. Finally, it discusses the relevance of these insights as a foundation for developing a follow-up interview-based qualitative study aimed at understanding how these elements are perceived by engineering students and how they shape ethical reasoning and moral intuitions among engineering students at TU Delft.

## Literature Review

Engineering ethics education in Europe developed at the intersection of three interconnected domains: its historical emergence as a field, the theoretical assumptions about technology and engineering that shaped it, and the learning outcomes and pedagogies through which it has been enacted. Thus, a brief account on the state of the art in each domain is necessary to provide the context of our research.

No systematic approaches to incorporating ethical training into engineering curricula emerged at the European level until the mid-1990s (Zandvoort et al., 2000, p. 296). “Systematic” refers to the coherent and consistent integration of such training within these programs. Inspired by earlier U.S. initiatives and prompted by a parliament law mandating the teaching of ethics in technical universities, the first milestones in Europe were reached in 1996 and 1998. In 1996, the Catholic University of Leuven and the Ethics Center in Lille started the European Ethic Network (EEN). This network produced the first handbook on engineering ethics (see Dubreuil & Goujon, 2000) with the intention of initiating a distinct European approach to engineering ethics. Hence, it included contributions from 10 European countries and presented a wide range of definitions and methodologies from engineers, ethicists, and philosophers of technology (Didier, 2000, p. 331). Although this handbook did not offer direct recommendations for integrating ethics training into engineering curricula, it played a role in clarifying the core conceptual assumptions of the field. In 1998, the European Society for Engineering Education (SEFI) established a working group on Ethics in Engineering Education. The main purpose of this group was to develop a broad framework for integrating ethics into engineering curricula, following the establishment of a wide-reaching network that brought together universities, professional associations, and industry partners through annual seminars and workshops (Porra, 2000, p. 337). Since then, SEFI has become the leading network for engineering education in Europe, significantly broadening its membership and range of activities.

Since the early stages of the development of this European approach to Engineering Ethics, its proponents held two assumptions. The first is that, along with providing technical solutions, engineers should raise questions concerning the ethical consequences of their professional practice and its impact on public interests. Although this may seem somewhat obvious, Christelle Didier remembered that at the time only Germany addressed these concerns, due to the hard lessons of World War II (Didier, 2015, p. 89). Second, to effectively raise these questions and arrive at meaningful answers, proponents of the European approach to engineering ethics highlighted the importance of considering the broader contexts of engineering practices, specifically, their social and institutional dimensions (Didier, 2015). In practice, this translated into a threefold approach to both the practice and teaching of engineering ethics, which contrasts with the dominant approach in American engineering ethics prior to the 1990s which was grounded in individualism and professional codes of ethics (see Didier, 2000, p. 331). It is worth noting, however, that American scholars were also beginning to challenge this individualistic tradition from within (see Davis, 1991; Herkert, 2005). Their work both paralleled and informed the emerging European debate.

Despite the assumptions underlying European engineering ethics programs that differed from their U.S. counterparts, the learning outcomes established by these European programs were inspired by the American approach, as highlighted by the Hastings Center in the early 1980s. As such, these outcomes specify the ethical competencies that engineering students are expected to acquire – competencies that remain largely relevant today, albeit with slight variations. To meet these goals, various teaching methods and pedagogical approaches have developed over the last three decades. On teaching methods, there has been a noted shift from the use of case studies and the application of ethical codes to more student-centered pedagogies, such as role-play exercises, gamification, student-initiated activities, and online educational activities (Polmear, 2025). Specifically, in terms of student-centered pedagogical approaches, five predominant strategies have come to the fore: (1) problem-based learning initiatives that expose students to real-world projects and situations; (2) value-sensitive design, which will be analyzed in this paper; (3) service learning, characterized by meaningful engagements between engineers and vulnerable populations that take place through a wide variety of humanitarian projects; (4) art-based and creative methods, including exercises such as creative writing workshops, maieutic discussions, and acting exercises; and (5) methods aimed at enhancing ethical reflection and fostering collective dialogue during the development of projects (Martin, 2025).

It is within this broader European context that TU Delft’s approach to engineering ethics education emerged and evolved, as the following sections document.

## Methods

This paper consists of a qualitative, integrative review (see Russell, 2005; Torraco, 2005). Integrative review is defined as a “form of research that reviews, critiques, and synthesizes representative literature on a topic in an integrated way such that new frameworks and perspectives on the topic are generated” (Torraco, 2005, p. 356). By critically reviewing and synthesizing various data sources on engineering ethics programs at TU Delft—including peer-reviewed journal articles, books, book chapters, conference papers, and website content—our goal was to conceptualize the historical, philosophical, and pedagogical foundations of the Delft approach to engineering ethics education. The findings from this review also provide contextual insights to inform the design of a follow-up, interview-based qualitative study on engineering ethics education at TU Delft.

This research addressed two questions: (1) What are the historical contexts and philosophical assumptions underlying the Delft approach to engineering ethics education? (2) What does this approach entail in practice, including learning outcomes and pedagogical methods? These questions are part of a larger comparative project on engineering ethics education across three cultural contexts—the United States, the Netherlands, and China—in which TU Delft serves as the primary institutional case for the European context.

The sources included 39 references in total, including 15 journal articles, 14 book chapters, 4 books, 2 conference papers, and 4 institutional documents and websites (Table 1). The selection process was purposive rather than exhaustive. Two coauthors with direct expertise on the research topic of this paper assembled an initial corpus. Such corpus reflected the published output of a core group of scholars from the Ethics and Philosophy of Technology section, which is responsible for teaching engineering ethics across all engineering programs at the institution. This group includes Andrea Gammon, Janna Van Grunsven, Jeroen Van den Hoven, Ibo van de Poel, Sabine Roeser, Udo Pesch, and Lavinia Marin among others. An initial search for published review articles on engineering ethics at TU Delft helped establish a preliminary structure, but revealed two gaps: the existing reviews were somewhat outdated and lacked a conceptual framework connecting the historical, philosophical, and pedagogical dimensions of the institution’s approach. Additional sources were incorporated to address these gaps—primarily works on the theoretical frameworks underpinning TU Delft’s approach, such as responsible research and innovation, design for values, and risk ethics, as well as methodological references on integrative review. The corpus is focused primarily on education-related publications, with the exception of sources consulted to analyze the theoretical assumptions section, which draw on broader philosophy of technology literature. The review covers both undergraduate and graduate education, though the latter appears only in relation to specific pedagogical exercises developed in recent years.

**Table 1:** Characterization of sources included in the integrative review overview of the 39 sources consulted in this review, organized by source type, time range, and focus area

Source Type	N	Time Range	Focus Area	Education Level
Journal articles	15	2000–2025	Engineering ethics education; philosophy of technology	Undergraduate and graduate
Book chapters	14	1980–2025	Engineering ethics education; theoretical frameworks (DFV, RRI, Risk Ethics)	Undergraduate
Books	4	2000–2010	Philosophy of technology; engineering ethics	N/A
Conference papers	2	2020–2021	Engineering ethics education—pedagogical exercises	Undergraduate
Institutional documents and websites	4	1998–n.d.	Institutional policies; accreditation criteria; software tools	Undergraduate
Total	39	1980–2025	—	—

*Note: Sources related to theoretical frameworks (DFV, RRI, Risk Ethics) draw on broader philosophy of technology literature beyond education-specific publications. The review covers graduate education only in relation to specific pedagogical exercises developed within the COMET project*

Throughout the entire process, the first author primarily led the analysis of sources. This process involved systematic reading, summarizing, and synthesis of the identified literature. Findings were then discussed and validated collaboratively with the remaining authors. Figures and tables were produced with the assistance of a generative AI tool, Claude Sonnet 4.6 (Anthropic), and subsequently reviewed and validated by the authors to ensure accuracy and consistency with the manuscript content.

## Results

The integrative review yielded findings along three dimensions. First, regarding historical context, the review identifies two distinct stages in the development of engineering ethics at TU Delft. Both mark an additive trajectory from compulsory stand-alone courses and traditional pedagogies toward modules within existing technical courses and the experimentation with experiential pedagogical approaches. Second, regarding theoretical assumptions, we show that TU Delft’s approach is grounded in a substantive conception of technology as value-laden, from which three applied frameworks derive: Design for Values, Responsible Research and Innovation, and Risk Ethics. Third, regarding instructional practices, we document a deepening and specification of learning goals across the two stages, alongside a series of innovative teaching exercises developed within the COMET project to put these goals into practice.

### Historical Context

TU Delft’s development of engineering ethics education reflects and in some ways pioneered the broader European trends outlined above. Table 2 summarizes the key events for the development of the programs of the institution. This development dates from 1993, when the Dutch Parliament passed the Bill of Higher Education and Research (Scheurwater & Doorman, 2001, p. 261). The Bill established that universities in the country had to include clear and explicit guidelines on the ethical aspects involved in their education and research activities (see Zandvoort et al., 2000, p. 299). In terms of implementing this directive, TU Delft was a forerunner, particularly among technological universities. In the same year, the Board of the university established an internal Advisory Committee of Ethics (ACE), with the aim of gathering recommendations to implement the Bill. In 1994, following the approval of the ACE’s recommendations, the Board directed the development of compulsory courses in ethics and engineering, to be designed and taught by the Faculty of Philosophy. In their pilot versions, these compulsory courses were called “Ethics and Engineering” and were first taught in 1996 (see van de Poel et al., 2001) in the faculties of Chemical Technology and Materials Science and Civil Engineering. The introduction of these pilot courses was successful. Within four years, these programs extended to other engineering degree programs. In practice, this meant that by the early 21st century, between 10 and 11 degree programs out of 15 or 16 offered by the university included this compulsory course (van de Poel et al., 2001; Scheurwater & Doorman, 2001).

**Table 2:** Key milestones in the development of engineering ethics education at TU Delft (1993–present)

Year	Key Events
1993	• Passing of the Dutch Bill of Higher Education and Research • Establishment of the TU Delft Advisory Committee of Ethics (ACE)
1994	• Approval of the ACE recommendations by the Board of TU Delft • The Board mandates the Faculty of Philosophy to develop a compulsory course in ethics and engineering following the ACE recommendations
1996	Beginning of the first pilot courses of ethics in the Chemical Technology and Materials Science and Civil Engineering faculties
2015	First pilot program of the Ethics Learning Lines (ELL) in undergraduate studies in the Civil Engineering faculty
2019	Launch of the COMET project to analyze, reconceive, and refine engineering ethics education practices in compulsory courses and the ELL

The table summarizes the key events in the development of engineering ethics education at TU Delft, from the passing of the Dutch Bill of Higher Education and Research in 1993 to the launch of the COMET project in 2019

TU Delft was not the only technological university to implement such programs. Institutions such as Eindhoven University of Technology, Wageningen University, and Twente University set up their own programs to comply with the Dutch Bill of 1993. However, TU Delft implemented these courses faster and with much more intensity. As Van de Poel et al. notes (2001, p. 268), in the early 2000s Twente University was just starting a professional ethics course for its Electrical Engineering degree and Eindhoven University approved the implementation of experimental projects in some non-specified degree programs.

The second milestone in the institution’s development started in 2015. In this year, the university launched the so-called Ethics Learning Lines (ELL, also referred to as “Ethics 2.0”) to complement compulsory courses (which TU Delft calls “Ethics 1.0”). Unlike the latter, the ELL are designed to be integrated into a wide variety of engineering courses outside the stand-alone ethics courses. The goal of the ELL is to intensify the collaboration between philosophy and engineering professors to enhance students’ ethical skills in ways that respond to the specific needs of their degree programs. The development of the ELL also reflects a concern that pedagogical approaches in the digital age should respond to the emerging needs of students from more diverse backgrounds—for example, neurodivergent learners. The development of the ELL also reflects a concern that pedagogical approaches in the digital age should respond to the emerging needs of students from more diverse backgrounds – for example, neurodivergent learners.

**Fig. 1:**
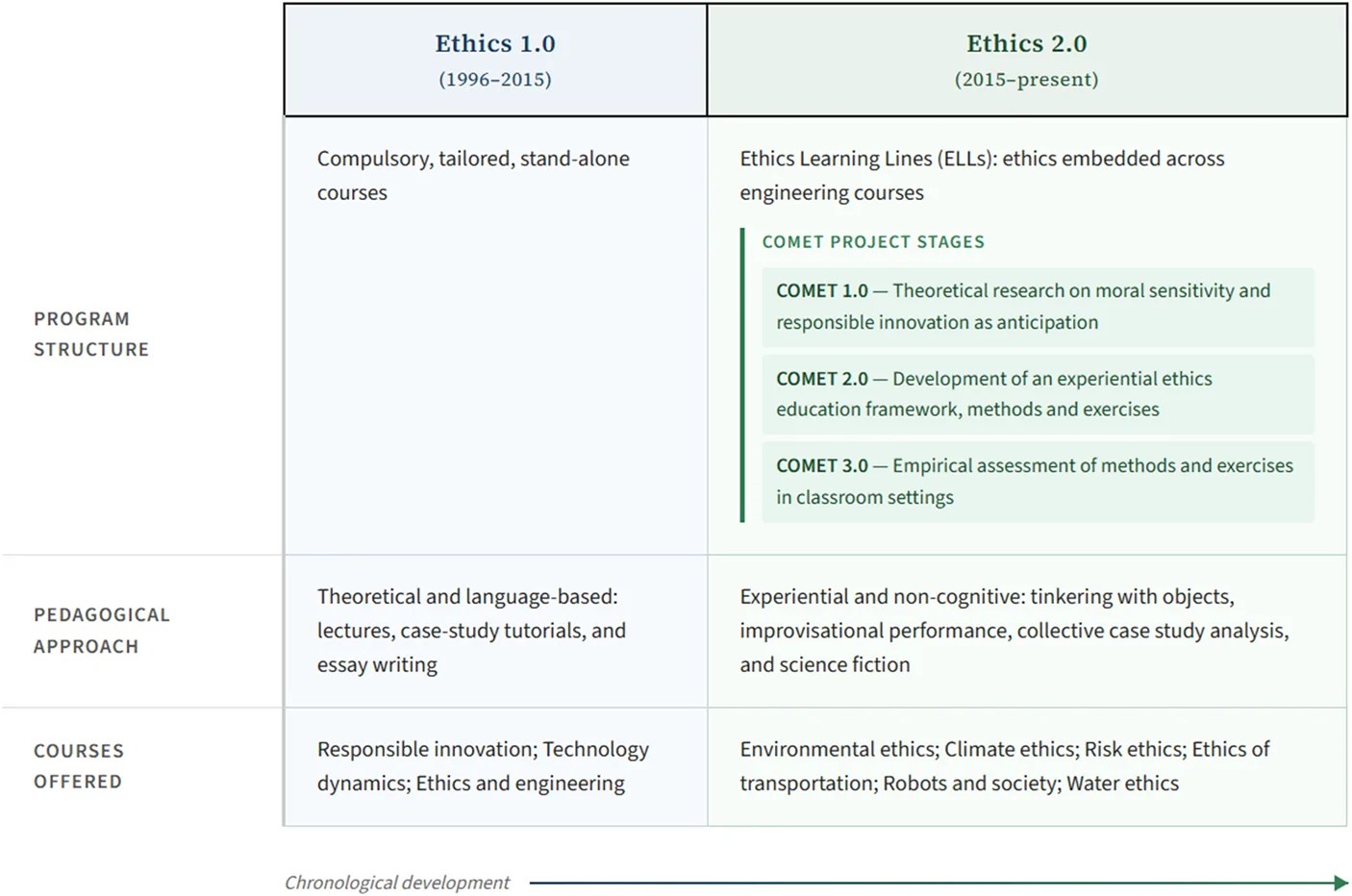
Contrast of the two stages of engineering ethics education at Delft University of Technology across three dimensions

To pursue these goals, the Comprehensive Ethics Teaching for Engineering and Design students (COMET) project began in collaboration with the rest of the 4TU federation. This federation is comprised of four technical universities: TU Delft, Eindhoven University of Technology, University of Twente, and Wageningen University. It aims to improve collaborations between research and public institutions and industry, enhance the international impact and societal relevance of research conducted within these institutions, and maximize the quality of training provided to engineers and designers (see 4TU. Federation, n.d.). The launch of the ELLs and the COMET project marks the beginning of what engineering ethics scholars at TU Delft have termed “Ethics 2.0” (see van Grunsven et al., 2021). This stage differs from Ethics 1.0 (see Fig. 1) primarily due to an experiential shift, reflected in engaging and innovative pedagogical methods and hands-on learning approaches.

The COMET project has three distinguishable stages. The first, called “COMET 1.0,” focuses on theoretical explorations of approaches to improve two moral skills: moral sensitivity and moral anticipation. (We re. to a fuller explanation of these goals in section 6.1.) During the second stage, called “COMET 2.0,” educators developed a more specific experiential ethics education framework—building on the theoretical explorations of COMET 1.0—and associated pedagogical exercises and methods to meet extended learning goals. In the third stage, called “COMET 3.0,” researchers apply the framework, methods, and exercises in classrooms, empirically assessing students’ learning outcomes and beginning to build theories about engineering ethics education based on these results. Understanding these initiatives requires first examining the theoretical assumptions and learning goals on which they are built.

### Theoretical Assumptions

**Fig. 2:**
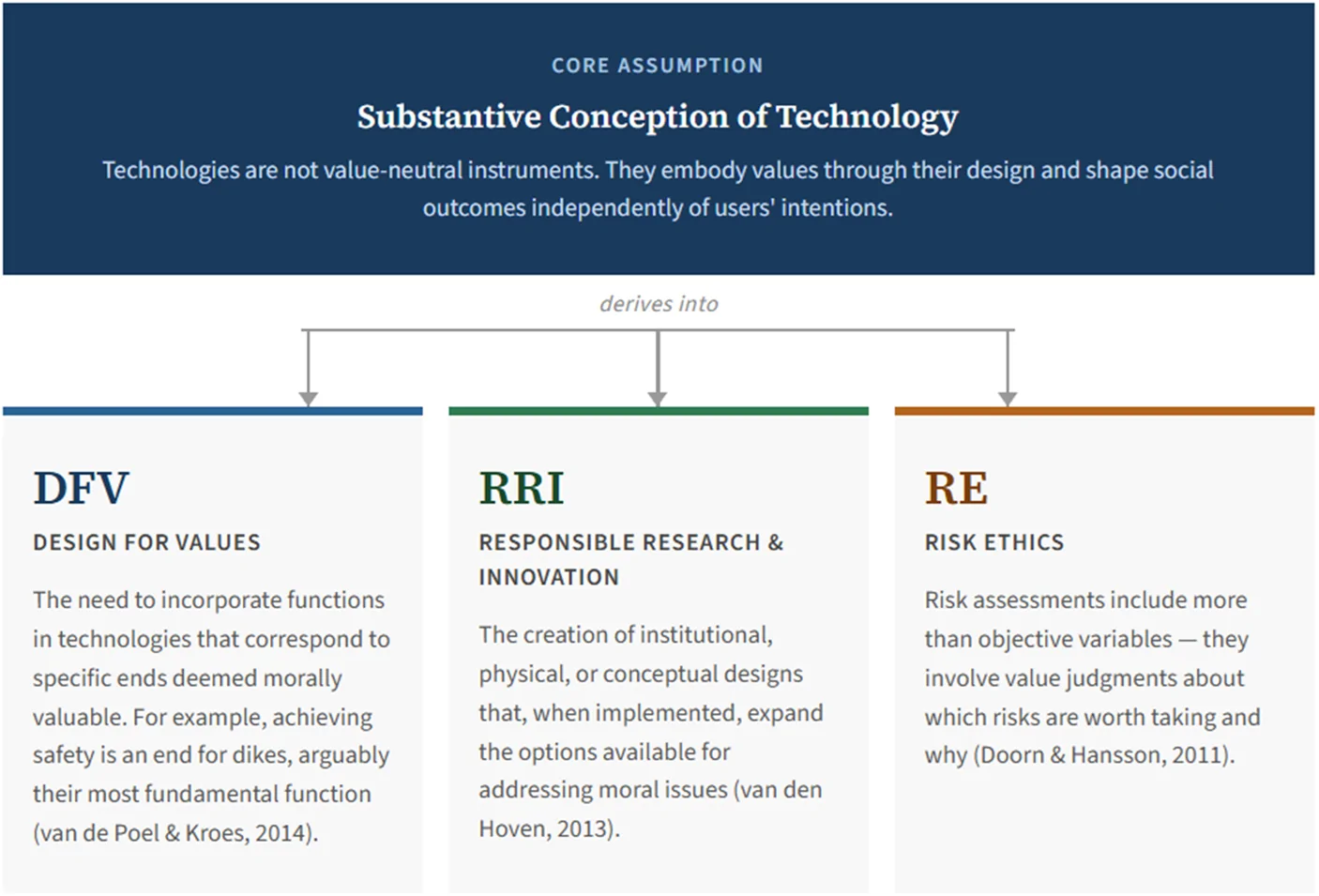
Theoretical assumptions of TU Delft engineering ethics programs

Engineering ethics programs at TU Delft are based on four core theoretical assumptions (see van Grunsven et al., 2021, pp. 3–4; Fig. 2). These assumptions inform the institution’s learning goals and pedagogical approaches. The core assumption is the so-called substantive conception of technology (see Feenberg, 2010, pp. 6–8; Rogers, 2008, pp. 38–40). Substantive theories of technology hold that technologies are not morally reducible to the intentions of their users. Therefore, technologies themselves are not merely instrumental or value-neutral, but value-laden. What exactly being value-laden means, however, differs. Some argue that technologies themselves can become moral agents due to their capacity to change human culture and societies (see Franssen et al., 2024). Others offer a weaker version, arguing that although only humans are moral agents, technologies can embody values in a specific way and under two conditions (see van de Poel & Kroes, 2014). Technologies, they argue, can embody final values that are not reducible to their physical properties but are nonetheless incorporated in their design—either because their ‘good making’ features directly reflect the value in question, or because such features can foster those values under the right circumstances. From this core theoretical assumption, engineering educators at TU Delft argue that being a good engineer involves developing not only technical but also ethical skills. Engineering, they contend, is not only about ‘getting something done’—practitioners also need to be aware of and take responsibility for the potential environmental, social, and political impact of the artifacts they design.

Building on the commitment to a substantive conception of technology, three more specific theoretical assumptions follow logically (van Grunsven et al., 2021, pp. 3–4): Design for Values (DFV), Responsible Research and Innovation (RRI), and Risk Ethics (RE). What they have in common is the idea that ethical issues and dilemmas are not ex post concerns—matters to be addressed only by decision-makers after a technology is designed—but ex ante responsibilities that engineers need to consider as an integral part of their professional practice. The underlying question is: What do engineers need to pay attention to in order to maximize the social benefits of their work? DFV, RRI, and RE offer complementary answers. The concept of DFV refers to the need to incorporate functions in technologies that correspond to specific ends deemed morally valuable. For example, as van de Poel and Kroes argue (2014, p. 114), achieving safety is an end for dikes, arguably their most fundamental function.

RRI refers to the creation of institutional, physical, or conceptual designs that, when implemented, expand the options available for addressing moral issues (van den Hoven, 2013, p. 82). Risk ethics involves assessing various scenarios in technological safety management, recognizing that such evaluations include more than purely objective variables and measurements based on mathematical probabilities—they also include value judgements about which risks are worth taking and the rationale behind these decisions (see Doorn & Hansson, 2011, p. 152). In sum, DFV and RE assume that since technologies can embody values, engineers need to consider them throughout the design process. Both DFV and RE also involve considering stakeholder interests and discourses, and the socially constructed nature of risk, as well as the potential consequences involved in designing and implementing technologies in specific cases. Meanwhile, RRI has received more attention from TU Delft scholars. (This feature will be discussed in the next section, on a novel framework for teaching engineering ethics based on the concept of “responsible anticipation” (Stone et al., 2020)).

To convey these theoretical assumptions to students and guide their professional formation, engineering educators at TU Delft follow a pragmatic approach. It seeks to avoid a common misconception in engineering ethics education: the perception that philosophers are simply prescribing behaviors or imposing values on future engineers (see van de Poel & Smuga-Fries, 2015, p. 214). The pragmatic approach has two distinct strands. First, engineering ethics courses are developed in collaboration with each engineering school, tailoring courses to the specific needs of each engineering discipline, without emphasizing highly abstract normative ethical frameworks. Second, rather than privileging abstract and non-contextual ethical thinking, each course follows a clear sequence of skills acquisition adapted to each stage of the ethical assessment processes that students, as future engineers, will need to navigate. This sequence is the “ethical cycle” (see van de Poel & Royakkers, 2011, pp. 133–160) that students are expected to become familiar with during engineering ethics programs. The cycle outlines a sequential framework consisting of five stages: moral problem statement, problem analysis, envisioning options for action, ethical evaluation, and reflection (van de Poel & Royakkers, 2011, p. 133). Each stage requires students to develop and exercise a specific ethical competence.

### Learning Goals

Following the ethical cycle model, engineering education programs at TU Delft aim to help students develop diverse practical ethical competencies (Table 3). Initially, these goals mostly followed the framework established by the Hastings Center in the United States in 1980 (see Callahan, 1980). Since then, however, instructors and researchers at TU Delft have further tailored these learning goals to address the specific needs of their own programs. First, they added context sensitivity to enhance the moral decision-making skills of students during the Ethics 1.0 stage (see van de Poel et al., 2001, pp. 270–271). In practice, such sensitivity assumes that engineers need to acknowledge the decision-making processes involved in their professional practice, beyond their individual circumstances (van de Poel et al., 2001, p. 269). For example, engineers might be constrained in their ethical assessments by corporate policies that are beyond their control. Second, in Ethics 2.0, instructors further specified the nature of the ethical competencies in their programs, including moral sensitivity, moral creativity, moral analysis, moral argumentation, moral judgement, moral justification, moral decision-making, and moral dialogue (Table 3).

**Table 3:** Learning goals of engineering ethics at TU Delft: past and Current

Ethics 1.0		Ethics 2.0
Initial Learning Goals (Hastings Center, 1980)	Updated Learning Goals (ABET EC 2000)	Emerging Developments
Recognition of ethical issues	Moral sensitivity	Universal care
Moral imagination	Moral creativity	Responsible anticipation: epistemic humility and reflexivity
Development of analytical skills	Moral analysis	*Moral analysis*
Moral obligation	Moral judgement	Ethical reflection
Tolerance and reduction of moral disagreement and ambiguity	Moral argumentation	Critical thinking
Context sensitivity	Moral dialogue	*Moral dialogue*
—	Moral decision-making	*Moral decision-making*

Note: Entries in italics indicate learning goals that remain unchanged across stages. “-” indicates no corresponding goal in the initial framework

The table traces the evolution of learning goals across three stages: initial goals drawn from the Hastings Center framework (Ethics 1.0), updated goals aligned with ABET EC 2000 criteria (Ethics 1.0), and emerging developments introduced in Ethics 2.0

As van de Poel & Royakkers point out (2011, p. 1), this change is mostly due to the need to follow the EC 2000 criteria established by the Accreditation Board for Engineering and Technology (ABET). Specifically, the learning goals aim to align with the third learning outcome in the ABET criterion, that engineering graduates should acquire “an understanding of professional and ethical responsibility” and “the broad education necessary to understand the impact of engineering solutions in a global and societal context” (Advisory Board for Engineering and Technology, 1998). Aside from the replacement of the so-called “moral obligation” goal (Table 3, Initial Learning Goals column), the differences between the ABET EC 2000 requirements and the original Hastings Center framework are, in practice, mostly terminological.

Moral sensitivity concerns the ability to recognize and identify ethical issues in everyday engineering practices. Both actions—recognizing and identifying—involve the rational articulation of moral responses to events or situations (see Callahan, 1980, p. 65). This rational articulation involves three processes (Mitcham & Muñoz, 2010), each contributing additional layers of cognitive complexity. The first is moral perception, or a preconscious intuition of a moral agent that someone may need their help and attention in a given situation. The second involves affectivity, or the empathic ability of a moral agent to put oneself in others’ shoes. The third is the assessment of so-called ‘dividing loyalties,’ which means the recognition and analysis of the stakeholders involved in diverse situations, along with the relationships that exist between them.

Building on these conceptual definitions, instructors at TU Delft during the Ethics 2.0 stage elaborated ways of adapting moral sensitivity for engineering students. These elaborations culminated in the notion of ‘universalized care’ (van Grunsven et al., 2023), according to which engineers can preserve and enhance their moral sensitivity in scenarios where there is significant physical distance between themselves and the people affected by the structures they create, maintain, or preserve. In practice, this means that engineers need to be aware of the ways their work affects collective well-being. To cultivate and support this awareness, instructors advocate for mechanisms that help bridge the physical gap, such as stakeholder consultations and client meetings.

The second learning goal is moral creativity, which refers to the ability to anticipate and analyze hypothetical ethical scenarios and dilemmas. More recently, TU Delft engineering educators paid special attention to this goal through a new framework, “responsible anticipation” (see Van Grunsven et al., 2024). This framework includes the following components: anticipation, reflexivity, epistemic humility, inclusion, and responsiveness. Anticipation enables engineers to imagine possible scenarios—what might happen and how they could proceed—while recognizing that this cognitive activity relies on underlying values. This reliance on underlying values is significant, instructors argue, to the extent that students need to be mindful of the way values shape anticipation practices. Students are expected to acknowledge biases and, therefore, to improve or change these values when necessary.

Doing so allows students to cultivate the second component of moral creativity: reflexivity. Reflexivity refers to an engineer’s mindfulness of their values when it comes to imagining the possible outcomes of technological innovation, and to what extent these values influence the way they exercise anticipation—particularly in exploring “what is plausible, likely or known” (Van Grunsven et al., 2024, p. 285). Instructors also emphasize epistemic humility, a virtue that requires awareness of the limits of an engineer’s knowledge, arising from the necessarily partial and situated nature of any individual perspective. Epistemic humility stands in contrast to epistemic hubris, often embedded in prejudices that hamper moral creativity. Examples include over-rationalist biases that undermine experiential knowledge due to ableist biases in design, or a displacement of emotions in moral anticipation processes. These biases assume that reason and emotions can be clearly separated. To illustrate this, instructors highlight the epistemic hubris underlying how engineers conceive the utility of exoskeletons—assuming they know what is best for disabled people without considering their perspectives and lived experiences.

Embracing reflexivity and epistemic humility leads naturally to a third component of responsible anticipation: inclusion. Inclusion here refers to overcoming what instructors call “epistemic injustices” that arise throughout the anticipation process. Engineering ethics instructors at TU Delft distinguish between two kinds of injustices. The first kind is testimonial, and it happens when agents directly affected by the creation and implementation of technologies are discredited as a reliable source of testimony due to racist, sexist, or ableist biases. The second kind is hermeneutic. It occurs when dominant concepts prevent marginalized people from making sense of and expressing their experiences in ways that are recognized, taken seriously, and trusted. To illustrate this concept, Van Grunsven et al. (2024) introduce the notion of technoableism, which emerges when technological innovations are assumed to remedy challenges faced by disabled people who are treated in a paternalistic way. Technoableism is often perpetuated when engineers select participants who confirm their perceptions, reinforcing what instructors call subtle forms of epistemic injustice (Van Grunsven et al., 2024, p. 291). Finally, engineering ethics instructors at TU Delft emphasize the importance of responsiveness, which refers to the capacity to change one’s values and perspective in light of the insights gained through the previously mentioned practices. Building on the substantive conception of technology, instructors argue that the most important step in cultivating responsiveness is abandoning the assumption that engineering practices and innovations are value neutral.

Although less emphasized in Ethics 2.0, the remaining learning goals are fundamental for introducing students to the ethical cycle. Moral analysis, which van de Poel and Royakkers define as the ability to examine moral issues by considering facts, values, stakeholders, and their respective interests within everyday professional practices (2011, p. 2), provides a good example. Moral judgement involves reaching ethical conclusions about issues and dilemmas by drawing on academic and non-academic ethical theories. Academic theories include virtue ethics, utilitarianism, Kantianism, and care ethics. Non-academic frameworks include professional ethics and common-sense morality. Moral decision-making consists of determining the appropriate course of action to address ethical issues and dilemmas after reflecting on relevant ethical theories or frameworks. Moral justification refers to the ability to explain how and why a particular ethical conclusion was reached, as well as the willingness to discuss and evaluate this reasoning with fellow engineers. Finally, moral dialogue extends this capacity to interactions with non-engineers, emphasizing the importance of engaging diverse perspectives in ethical deliberation

In terms of how these teaching goals can be taught, instructors emphasize the value of ethical reflection and critical thinking. Through exercising both skills, engineers learn how to decide what to believe and how to act—recognizing and mitigating biases while attending to the contextual specificities of their professional practice. As Marin argues (Marin, n.d.), the difference between critical thinking and ethical reflection lies in the domains to which they are applied. While critical thinking usually applies to empirical questions—such as determining the truth or falsity of statements and arguments—ethical reflection concerns normative issues. In such debates, the focus shifts to what we ought to do and, thus, factual claims are not the primary subject of evaluation.

### The “Experiential Turn”: Emerging Pedagogical Initiatives

In its early years—the Ethics 1.0 era—the pedagogical approach to mandatory engineering ethics courses followed a traditional lecture-and-tutorial format. Lectures were designed to provide the theoretical background necessary for undergraduate engineering students to develop their ethical competencies. These competencies were then applied in tutorials where students engaged with case studies. In the mid-2000s, instructors introduced a complementary tool to enhance both lecture content and tutorial discussions: the Agora computer software (see Van Der Burg & Van De Poel, 2005). The goal of Agora is to reinforce the material taught in compulsory courses through a range of interactive exercises. Each exercise aligns with a stage in the ethical cycle, allowing students to practice and internalize specific ethical skills through virtual case studies. Nowadays, Agora is widely used not only at TU Delft but also at other Dutch technical universities—University of Twente and Eindhoven University of Technology—and internationally, including the Kanazawa Institute of Technology in Japan (Eindhoven University of Technology, n.d.).

In recent years, TU Delft engineering educators have developed alternative pedagogical methods to complement traditional lectures and tutorials. The overarching aim of these pedagogical innovations is to make engineering ethics more responsive to emerging societal realities and the diverse needs of today’s students. Two milestones illustrate this recent turn. We use the term “turn” simply to describe a shift in teaching approach, not to suggest that it produces better outcomes. The first is the inclusion of the Ethics Learning Lines (ELLs) in engineering courses, with a particular focus on enhancing students’ moral sensitivity, as discussed above. The second takes an ‘experiential turn’ in engineering ethics education, with the goal of providing more inclusive and responsive learning environments. This experiential turn holds that future engineers can better acquire ethical skills by directly engaging with ethical dilemmas and problems rather than through exclusively text-based approaches such as essay writing and text analysis. In practice, this involves a pedagogical evolution from the writing-assignment approach to the use of creative and performative methods to cultivate ethical reflection. Examples of this experiential turn include four exercises.

A first exercise has been used in the environmental ethics course for master’s students. Its purpose is to enhance their moral sensitivity and critical thinking skills. The idea is to collectively analyze specific case studies during class sessions – for example, the implementation of low-traffic neighborhoods to promote alternative means of transportation (see Gammon & Marin, 2022). The most important aspect of this exercise is that students not only acquire theoretical knowledge but are also exposed to specific situations that allow them to practice moral sensitivity. As Gammon and Marin point out, the exercise consists of three steps. First, students are asked to identify the stakeholders involved in the case study – in this instance, the various agents connected to low-traffic neighborhoods – and to articulate their interests. Second, students practice moral sensitivity by responding to a set of guiding questions, such as: Does the establishment of low-traffic neighborhoods address an environmental problem? Why? Is this primarily an ethical or a political issue? Why? Finally, the class is split into different groups to discuss the technical, political, ethical, and environmental dimensions of the issue. Each group is also tasked with imagining solutions. These ideas are then shared and discussed collectively during the session.

The second is a pedagogical exercise: Tinkering with objects to explore potential improvements (see Van Grunsven, Franssen et al., 2024). This exercise has two major goals: (1) creating more inclusive learning environments, with a special focus on neurodivergent students—such as those with ADHD, dyslexia, or autism—whose ways of learning and engaging often differ from traditional pedagogical methods, such as lectures, text-based instruction, and writing assignments; and (2) enhancing students’ moral sensitivity and reflective capacities by challenging their assumptions about ableism and accessibility in the design of technologies and artifacts. The exercise was tested in three courses, in 3-hour workshops in which students engaged in two rounds of tinkering with pre-assigned objects. During the second round, students received feedback from other groups. To assess the effectiveness of this pilot exercise – i.e., to what extent the exercise was able to create more inclusive learning environments, and to call into question assumptions about ableism and accessibility – instructors and researchers conducted a poll, three semi-structured interviews (to gather students’ feedback on their learning experiences and suggestions for improvement), and ethnographic observations during the workshops. Overall, the first goal was achieved, while the second is still in progress.

A third exercise, implemented in honors programs, is the use of improvisational performances to enhance students’ moral sensitivity. The aim was to push beyond the limits of traditional role-playing exercises, where students often lacked personal attachment to the scenarios they were enacting, resulting in the replication of stereotypical roles. The first trial of the exercise took place during the autumn-winter semester of 2020 (Marin et al., 2021, p. 358). The exercise consists of the following. Once students are taught basic improvisational performance skills, they are asked to perform in groups of three. Each student plays a distinct role in a performance focused on imagining a scenario in which engineering devices can speak. One student plays the role of the engineer, another plays the speaking object, and the third plays the person who benefits from the device. To assess the effectiveness of the exercise in enhancing students’ moral sensitivity, a surprising finding emerged. The unexpected finding was that the students’ ability to identify ethical issues improved not through performing themselves, but also by observing their peers perform. In other words, improvisational performances had indirect yet valuable educational benefits. Marin et al. (2021) identify two effects to account for these results. The first is the spectator effect: students became more adept at recognizing ethical issues when observing their peers perform than when performing themselves. The second is the shared space of vulnerability effect: both performing and witnessing improvised scenarios cultivated a sense of shared vulnerability, encouraging students to acknowledge their emotions and personal limitations, and thereby enhancing their moral sensitivity and engagement

This experiential turn, however, does not mean that instructors have completely abandoned traditional approaches. Evidence of this balance can be seen in the fourth exercise: Collective learning and the practice of moral imagination through science fiction. Combining essay writing with collective deliberation, this exercise illustrates the kind of empirical work being developed within COMET 3.0, in which pedagogical methods are systematically tested and assessed to build theory about engineering ethics education. As such, the goal of the exercise is to enhance students’ ability to consider and, when appropriate, integrate multiple perspectives in addressing moral dilemmas. Students engage with a single hypothetical scenario by writing two essays—one before and one after participating in the exercise. The aim is to evaluate how other students—feedback and exposure to diverse viewpoints influence students’ reasoning and ethical framing of the scenario.

The assessment of the learning outcomes of this exercise took place in the four following stages:

1. Pre-exercise stage: Instructors and researchers surveyed students about how many solutions they could envision for the scenario and how many they considered morally acceptable.
2. Post-exercise stage (survey): The same survey was administered after the exercise, with students asked to justify any changes in their answers.
3. Post-exercise stage (open-ended survey): Students were asked whether they had revised their essays and, if so, to explain why.
4. Essay analysis and interviews: Instructors compared the pre- and post-exercise essays. When changes were observed, some students were interviewed to understand the reasons behind their revisions.

It should be noted that systematic reporting of learning outcomes across these exercises remains limited at this stage. Moreover, these exercises are not designed to evaluate whether Ethics 2.0 produces better learning outcomes than Ethics 1.0. Their purpose is to explore and develop innovative pedagogical approaches responsive to the needs of today’s engineering students, not to establish comparative effectiveness. With the exception of the improvisational performance exercise, the empirical assessment of whether these approaches effectively achieve their stated learning goals is ongoing. This is precisely the research agenda that COMET 3.0 is designed to address, and it constitutes one of the central motivations for the follow-up qualitative study proposed in this paper.

In summary, teaching at TU Delft has evolved from traditional lectures and tutorials to more innovative, experiential approaches aimed at enhancing the learning goals described above. While foundational tools like the Agora software continue to support case-based learning, newer methods—including collaborative case analysis, object-based tinkering, improvisational performances, and speculative scenarios using science fiction—prioritize inclusive, reflective, and non-cognitive learning experiences. These pedagogical innovations reflect a broader experiential turn that complements, rather than replaces, existing pedagogical practices.

## Discussion: Implications for a Follow-Up Interview-Based Qualitative Study

An important contribution of this integrative review is to explore if and how the identified themes shape students’ experiences of ethics learning at TU Delft. Throughout this review, we have reconstructed TU Delft’s approach largely as it is articulated by its instructors and scholars. Our aim has not been to endorse this framework uncritically, but to analyze its underlying assumptions, internal coherence, and pedagogical evolution. By making these layers explicit, we create space for comparative reflection and critical engagement, both within TU Delft and across other institutional contexts. To build on this review, we envision a follow-up qualitative study involving semi-structured interviews with engineering students who have completed courses that incorporate the ELLs. This interview-based qualitative study will be guided by three sets of questions: (1) To what extent, and in what ways, do engineering students at TU Delft understand and agree with the four core assumptions, particularly the foundational view that technologies are value-laden? (2) Are students aware of the seven ethics learning goals, and how do they prioritize these goals? (3) How do students perceive the experiential and engaged approaches to engineering ethics education introduced in these courses?

The first set of questions relates to the longstanding debate on the moral status of technologies, which centers on two opposing positions: the value-laden and the value-neutrality assumptions (see Franssen et al., 2024). The value-laden view argues that technologies possess moral significance in and of themselves, and thus cannot be fully reduced to the intentions or goals of their users. This is because technologies exhibit properties that can promote certain values or outcomes independently of their users’ intentions. For example, Amory Lovins (1979) characterized ‘soft path’ energy infrastructures—such as those based on solar and wind power—as inherently aligned with political decentralization, in contrast to centralized fossil fuel or nuclear power systems. On the other hand, the value-neutrality view (see Pitt, 2014) holds that technologies do not inherently embody values. Instead, their moral significance depends entirely on the diverse—and often conflicting—purposes of the human agents who use them. The classic illustration of this position is the knife: a tool that can be used for a range of morally divergent actions, such as cutting a rope or committing a violent act, but that carries no intrinsic moral orientation on its own.

As Miller (2021) notes, the value-laden position currently dominates contemporary philosophy of technology. However, our first hypothesis is that this dominance may not extend to engineering students at TU Delft, particularly those without prior ethical training. To explore this, we aim to investigate the following research questions: (a) Does the value-laden perspective, as held by most TU Delft instructors of engineering ethics, resonate with students? More specifically, do students agree with the idea that technologies can embody values through their design functions? (b) Do students’ views on this issue shift over the course of completing ELL-embedded courses?

We also aim to explore student perspectives on two further theoretical assumptions underlying TU Delft’s approach: RRI and risk ethics. Regarding RRI, our focus is on examining the extent to which students accept the view that being a good engineer requires both technical and ethical competencies. Specifically, we seek to address the following research questions: (a) Do students believe that engineers should engage with ethical questions in their daily professional practice as often as they engage with technical challenges? (b) Or do they think such ethical considerations should primarily be handled by policymakers and applied ethics experts? (c) Do students’ views on these issues shift after completing courses? We hypothesize that while students acknowledge the importance of ethical skills, they may still view them as secondary to technical expertise

In terms of risk ethics, we are particularly interested in how students understand the nature of risk in the design and implementation of technologies and infrastructures. Our focus is on the potential tension between probabilistic risk assessments and value-laden judgments about risk. For instance: (a) Would students support the construction of an infrastructure project when there is a conflict between probabilistic risk assessments and public perceptions or concerns about safety? In such cases, do students prioritize formal risk models or the lived experiences and opinions of affected stakeholders? (b) Do students’ views on this issue change over the course of ELL-based instruction?

Turning to the second set of questions, we aim to explore how students perceive the five-step approach to engineering ethics decision-making in relation to their future professional practice. Specifically: (a) How do students assess the usefulness of the five-step method for improving their professional performance? (b) Do they see it as a valuable tool for improving ideation and design processes, or do they view the ethical competencies it cultivates as secondary or irrelevant to day-to-day engineering work? In relation to one of the oldest pedagogical concerns raised by TU Delft instructors, we also ask: (c) Do students feel that the five-step approach helps them become more autonomous in ethical reasoning, or do they still experience it as a prescriptive framework tied to philosophical authority? (d) Are students satisfied with the level of theoretical content embedded in this pragmatic approach, or would they prefer a more robust theoretical foundation to support the practical strategies offered for addressing moral dilemmas in engineering?

We also aim to investigate how students engage with epistemic hubris—defined earlier as the over-rationalistic bias that hampers moral creativity—particularly regarding the relationship between reason and emotion in ethical decision-making. This leads to the following questions: (a) Do students believe that emotions play a more significant role in ethical reasoning than is typically acknowledged when reason and emotion are treated as separate domains? (b) Do they find that incorporating emotions into ethical reflection enhances their professional performance and ability to serve society, or do they think that a clearer separation between reason and emotion is preferable for fulfilling these responsibilities?

Turning to the third set of questions, we aim to address several issues related to experiential learning in engineering ethics. The first concerns ableist biases in technology design, recently examined by engineering ethics instructors at TU Delft (see Van Grunsven et al., 2024), who identified the persistence of such biases even after teaching interventions involving hands-on activities like tinkering with experimental devices. It is therefore important to investigate whether these biases persist more broadly—not only among students who have participated in experiential exercises. To address this concern, the semi-structured interviews will include questions such as:

(a) How do students perceive the humanitarian goals of exoskeleton design and development, particularly in relation to people with disabilities? Do they view these devices as intended to “rescue” disabled individuals from a perceived “disgrace”? (b) Would students seek the perspectives of disabled individuals about their daily experiences if they were tasked with designing similar technologies?

The second issue concerns students’ attitudes toward the non-cognitive dimensions of ethical learning and their perceived role within the courses. To what extent do students believe that teaching activities involving non-cognitive, or at least not exclusively cognitive, skills—such as tinkering or improvisational performance—effectively reinforce the knowledge acquired during the course? Are students inclined to regard these activities as merely temporary, somewhat “trivial breaks” before engaging with “more serious” tasks like essays or exams? In other words, have these exercises shifted students’ views on whether such activities complement theoretical instruction or are simply forms of entertainment? Furthermore, have students’ opinions changed over the course of their ethics education?

## Conclusion

In the increasingly globalized field of engineering ethics education, TU Delft stands out as a pioneering and continually evolving institution in Europe. This paper has offered a qualitative, integrative review of the literature related to how instructors at TU Delft approach and teach engineering ethics. Given that these students are likely to play critical roles in developing technical solutions to address pressing societal challenges, it is practically significant to examine the underlying assumptions, learning objectives, and pedagogical approaches that shape their ethical education.

The review reveals a coherent and evolving institutional approach built on three interconnected layers. The first is a foundational philosophical commitment—the substantive conception of technology as value-laden—from which three applied frameworks derive: DFV, RRI, and Risk Ethics. The second is a set of learning goals that have deepened over time, moving from the broad competencies established by the Hastings Center toward more specific ethical skills—including moral sensitivity, moral creativity, and moral dialogue—tailored to the needs of engineering students. The third is a pedagogical evolution from traditional lectures and tutorials toward experiential and non-cognitive approaches that engage students as active moral agents rather than passive recipients of ethical knowledge. Drawing on the conceptual framing offered by instructors—while also engaging with it critically—we have shown that these three layers are not independent but mutually reinforcing, forming what TU Delft scholars have termed Ethics 2.0. Finally, we have argued for the relevance of this review as a foundation for a follow-up qualitative study aimed at exploring how these components are perceived and interpreted by engineering students at TU Delft.

Beyond these specific findings, this paper’s contributions speak to three broader debates in engineering ethics education. The first concerns pedagogy. By documenting TU Delft’s experiential turn in detail, this paper provides an empirically grounded reference point for the normative debate about how ethics should be taught to engineers. It also offers a benchmark for future comparative work assessing what experiential and non-cognitive approaches concretely add to students’ ethical formation relative to traditional methods. The second concerns curricular integration. Specifically, should ethics be taught as a standalone discipline or embedded within technical courses? TU Delft’s model, which combines both approaches through Ethics 1.0 and Ethics 2.0, offers a concrete case for examining this question. The institutional evolution documented here suggests these approaches are not mutually exclusive. However, the conditions under which their combination is effective remain an open question for other institutions to examine. The third concerns the moral status of technology. TU Delft’s explicit curricular commitment to the value-laden position raises the question of whether ethics programs should take a first-order stance on whether technologies embody values, or whether curricula are better served by a second-order approach - one that develops students’ capacity to reason across competing positions rather than from within one. This is not merely a philosophical question. It has direct implications for how learning goals are formulated, how case studies are framed, and how students with different prior assumptions are engaged. While the specific findings described here are institutionally situated, the three-layer structure we identify, philosophical assumptions, learning goals, and pedagogical practices, offers an analytic lens that other institutions can use to examine and reflect on their own engineering ethics programs.

Building on these broader contributions, we propose three avenues for further research. First, as discussed earlier, we propose conducting a follow-up qualitative, interview-based study to explore how TU Delft students perceive engineering ethics education after completing the ELL-embedded courses. This empirical investigation would be essential for assessing the effectiveness of the ELLs as a defining feature of Ethics 2.0. Second, drawing on the comparative dimension of the larger project of which this study is part, we suggest conducting comparative empirical research focused on how cultural differences between countries might shape students’ perceptions of the teaching methods, pedagogical exercises, theoretical assumptions, and learning goals discussed in this paper. Third, we advocate for an expanded review of how other institutions approach the teaching of engineering ethics, with the goal of identifying both commonalities and differences across diverse national and cultural contexts. From a theoretical perspective, it would be particularly useful to explore how the assumption of value-neutrality in technology is viewed in non-Western cultural settings. This could help assess whether the instrumentalist view of technology, long critiqued by philosophers of technology such as Heidegger and Ellul, remains a particular Western cultural construct.

## Data Availability

The data for this paper consist of published journal articles, conference proceedings, book chapters, books, and webpages. No new data were generated for this study.
